# A Qualitative Approach to Understanding the Holistic Experience of Psychotherapy Among Clients

**DOI:** 10.3389/fpsyg.2021.667303

**Published:** 2021-08-06

**Authors:** Lee Seng Esmond Seow, Rajeswari Sambasivam, Sherilyn Chang, Mythily Subramaniam, Huixian Sharon Lu, Hanita Ashok Assudani, Chern-Yee Geoffrey Tan, Janhavi Ajit Vaingankar

**Affiliations:** ^1^Research Division, Institute of Mental Health, Singapore, Singapore; ^2^Department of Psychology, Institute of Mental Health, Singapore, Singapore; ^3^Department of Moods and Anxiety, Institute of Mental Health, Singapore, Singapore

**Keywords:** psychotherapy, qualitative, intervention, holistic, service utilization

## Abstract

**Background:** The study of the experience of clients across multiple service encounters (or touchpoints) is important from the perspective of service research. Despite the availability of effective psychotherapies, there exists a significant gap in the optimal delivery of such interventions in the community. Therefore, the aim of this study was to explore the experience of psychotherapy among clients integrating the before–during–after service encounters using a qualitative approach.

**Methods:** A total of 15 clients of outpatient psychotherapy were interviewed, and data saturation was reached. The topics included pathways and reasons to seeking psychotherapy, aspects of the therapy process that have been helpful or unhelpful, and perceived change after receiving psychotherapy. Information was analyzed using the inductive thematic analysis method. Emergent themes pertaining to pre-psychotherapy encounters were mapped onto major components that were identified in Andersen's Health Service Utilization Model.

**Results:** Mental health stigma and the lack of understanding about psychotherapy were the predisposing factors that impeded service use while the preference for non-pharmacological intervention promoted its use. Enabling factors such as affordability and service availability were also of concern, along with perceived and evaluated needs. The attributes of therapists, application of techniques, and the resistance of the client were found to impact the therapeutic alliance. While the majority of the clients experienced positive change or had engaged in self-help strategies after receiving psychotherapy, some cited limited impact on the recovery of symptoms or problematic self-coping without the therapists.

**Conclusion:** This study proposes to expand on Andersen's Behavioral Model by including therapy-related factors so as to provide a more holistic understanding of the use of psychotherapy among the clients. More importantly, the study identified several barriers to access and negative experiences or outcomes, which should be addressed to promote uptake of the psychotherapy intervention.

## Introduction

The “person-centered” approach to care delivery has been valued as a core part of service design and is necessary to provide a nurturing environment that is respectful, compassionate, and responsive to the needs of the individuals. Understanding the situation of or lived experience of health services by each client has increasingly been recognized as a key element of quality healthcare to improve safety and patient outcomes. For example, patient satisfaction with service or effectiveness of interventions promotes treatment compliance and supports recovery in mental health settings (Katsakou et al., 2010; Urben et al., 2015).

Psychotherapy, also called “talk therapy,” is a process by which the emotional and mental health-related problems are treated through communication and relationship factors between an individual and a trained mental health professional (Herkov, 2016). Despite clear evidence for the efficacy and effectiveness of psychotherapy in general (Lambert, 2013b), about 35–40% of patients experienced no benefit while a small group of 5–10% experienced deterioration in their condition on completing treatment in randomized clinical trials (Hansen et al., 2002). In a routine practice where treatments averaged four sessions, the rate of improvement was reported to be only about 20% (Hansen et al., 2002). Findings from numerous studies also estimated around 25–50% of patients across diverse treatment settings to “refuse psychotherapy” by failing to return to treatment after initial intake or therapy session (Garfield, 1994). The premature termination of sessions has been a problem that hinders the effective delivery of psychotherapeutic treatment as many patients tend not to receive the “adequate dose” of therapy, which is required for them to observe the desired symptomatic relief (Hansen et al., 2002; Anderson, 2016). Furthermore, such attrition or no-show wastes mental health resources and staff time, denies access to those in need, and limits the ability of organization to serve those in need (Joshi et al., 1986). Compared with those who completed treatment courses, those who defaulted are usually less satisfied with services (Lebow, 1982). Prior studies have shown that the optimal way to predict treatment outcome is to measure their distress pretreatment (Lambert, 2013a). The predisposing factors are those that increase his/her inclination to health service use and may include characteristics such as demography (i.e., age and gender), social structure (i.e., education, occupation, ethnicity, social interactions, and network), and health beliefs (Andersen, 1995; Andersen and Newman, 2005). Quantitative studies have also consistently revealed being female, single or divorced, unemployed, and having a higher education level to be significantly associated with the use of psychotherapy (Olfson and Pincus, 1994; Briffault et al., 2008; Hundt et al., 2014) or the service utilization of mental health (Parslow and Jorm, 2000; Roberts et al., 2018; Ayele et al., 2020).

From the perspective of service research, the experience of the client is conceptualized as a “journey with a service provider over time during the service utilization cycle across multiple touchpoints” (Lemon and Verhoef, 2016). Psychotherapy service research has usually focused on understanding, measuring, and optimizing the in-session experience or the treatment process of the client, but what happens leading up to the intervention and after the intervention has received less attention. The narrowed focus on the delivery of the core service itself has prevented service researchers from recognizing the evolving needs of client for a holistic service experience, which spans all potential service encounters (Voorhees et al., 2017).

Therefore, this study aimed to address this gap by integrating “pre-therapy,” “during therapy,” and “post-therapy” service encounters to gain an in-depth understanding of the experience of the clients of using psychotherapy services. In doing so, we hoped to identify help-seeking pathways, as well as positive and negative experiences or outcomes from the service engagement of client, and to discuss any policy implications with respect to these findings.

## Methods

### Participants, Recruitment, and Setting

This study was conducted among individuals attending outpatient psychotherapy at the Institute of Mental Health, a tertiary psychiatric hospital in Singapore. Participants were recruited using a mix of personal network and purposive sampling. The majority of patients were referred by mental healthcare professionals (e.g., psychologists and clinicians) who provided psychotherapy services in the institute. Posters were also placed in the clinic to inform the clients of the ongoing study with information on the eligibility criteria and the contact of researchers was provided for self-referral by patients. The inclusion criteria were those who were aged 21 years and above, those who were able to provide consent, and those who had attended at least two psychotherapy sessions in the past year. All participants provided written informed consent and were given a token sum for their time upon completion of the study. The approval of the study was obtained from the institutional ethics committee, the Domain Specific Review Board of National Healthcare Group, Singapore (DSRB Ref No: 2018/00870). Interviews and recruitment of new participants continued until the study achieved data saturation, which was determined by the repetition of themes or subthemes (i.e., no new information was evident). A total of 15 participants were therefore enrolled from the period of January–October 2019.

### Study Procedures

This interpretative qualitative study was a part of a bigger study that aimed to understand the psychotherapeutic strategies and interventions to improve positive mental health among psychotherapy clients. Participants were first asked to self-complete a short questionnaire to obtain information on the sociodemographic background (e.g., age, gender, education, and occupation) and clinical history (e.g., diagnosis, age of onset, hospitalization, and the number of psychotherapy sessions). In-depth interviews were then conducted by a facilitator (JV or SC) at mutually agreed places using a common interview guide to ensure standardization across the participants. The interview schedule was designed to allow a free exchange in the discussion, guided by the narrative of the participants. Participants were first asked about their background in terms of their family, work, diagnosis, onset, and symptoms, as well as their recent experience with psychotherapy, and were encouraged to describe in detail. Probing questions served as prompts to elicit a richer understanding and were found in the interview guide (Table 1) to ensure that the data collected across the sessions would be as uniform as possible.

**Table 1:** Interview guide.

Can you please tell me about your recent experience with psychotherapy? **Probes** Why did you decide to take it up? Who referred you? What was your main concern? How long/how many sessions have you had so far? What has been your experience like? Have things changed for you since you received therapy? Have there been times when psychotherapy improved your psychological well-being? What has been helpful in those times? What has not been helpful?

### Data Analysis

**Figure 1:**
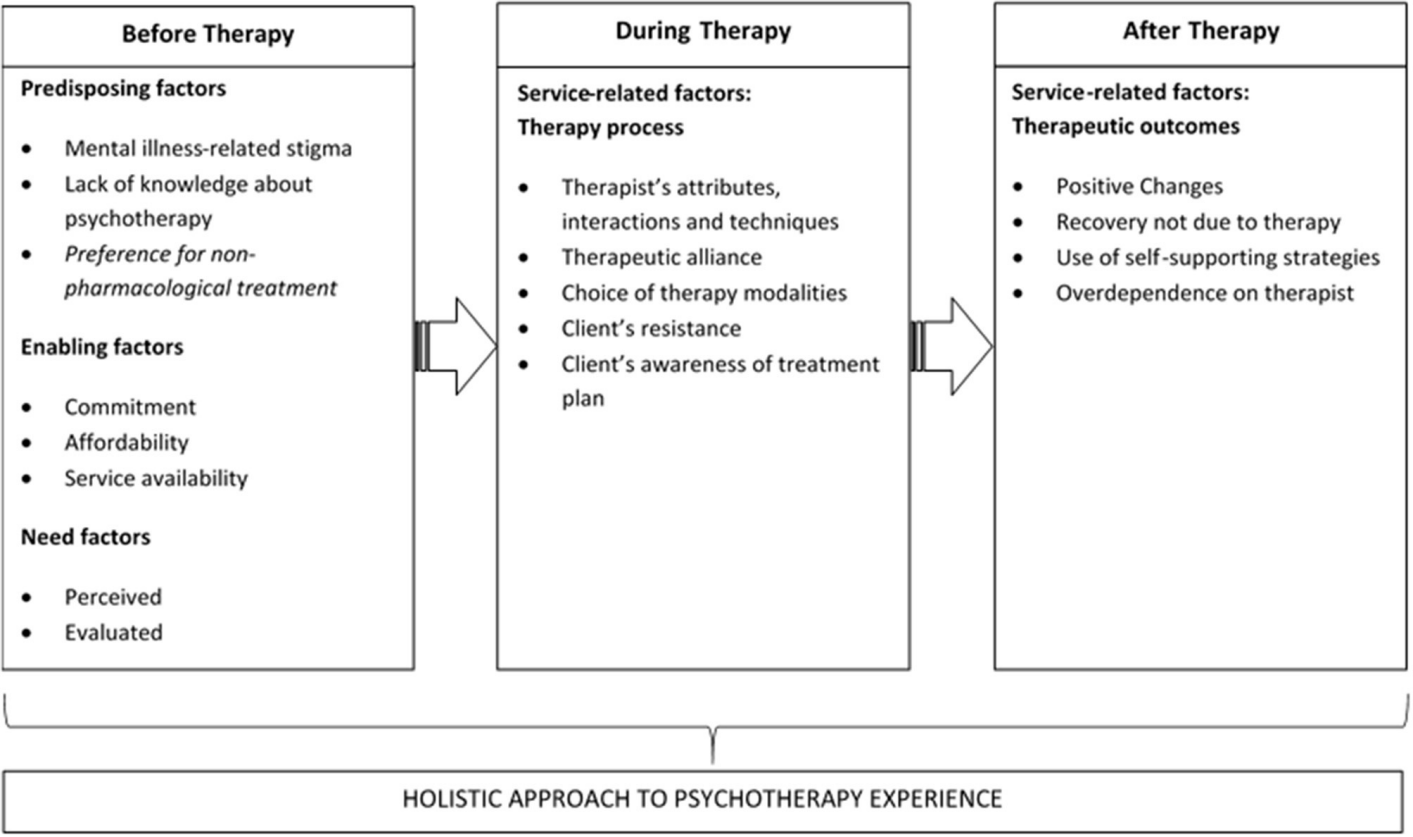
Client experience with psychotherapy service utilization.

All interviews were audiotaped and transcribed verbatim, with transcripts checked for consistency by another team member. NVivo software version 11 was used for the purpose of coding and data processing (QSR International; Computer Software, Australia). The data were analyzed using the thematic analysis that involved discovering, interpreting, and reporting patterns and clusters of meaning within the data (Braun and Clarke, 2006). In the first step, all study team members (JV, SC, ES, and RS) independently read a transcript each and employed either descriptive or theoretical codes to index meaningful segments or contents. The next step involved gathering of the team to compare individual analyses, reconcile any differences of perspective, and achieve consensus on the codes and their themes. From this initial inductive coding scheme, a list of preliminary themes was generated based on the summaries and collective interpretation of the coded material. To confirm adequate inter-rater reliability, a codebook was then constructed and all members coded a single new transcript using the codebook as a guide. Cohen's kappa coefficient was established to be 0.83, and team members proceeded to code the remaining transcripts independently. To capture unexpected themes that emerged during the course of reading the remaining transcripts, additional codes were created through open coding. In the final step, all identified themes were progressively integrated into higher-order key themes in relation to the research topic. To differentiate the before–during–after periods of service encounters, we have organized our findings into three distinct sections, namely, pre-, during-, and post-psychotherapy (Figure 1).

### Thematic Mapping to Conceptual Framework

Our analysis was underpinned by Andersen's Health Service Utilization Model (Andersen, 1995), which has been used extensively in studies to understand factors that both promote and undermine the access to healthcare. Findings pertaining to the pre-psychotherapy experience provided support for the model, particularly where emergent themes relating to pathways and reasons to help-seeking could be mapped onto major components identified in the model. Andersen's Behavioral Model defined health service use as an interplay of three distinguished determinants, namely, predisposing characteristics, enabling resources, and need factors (Andersen, 1995; Andersen and Newman, 2005). The predisposing characteristics refer to the sociocultural characteristics of the individuals that exist prior to the development of an illness. The enabling factors represent the logistic aspects of obtaining care such as affordability and availability of resources at the personal, family, and/or community level. Need factors, usually identified as the most immediate cause of health service use, include potential needs for care, perceived and evaluated health, or functional state. We adopted thematic mapping onto an existing framework and discussed themes associated with each determinant to present our findings with respect to the pre-psychotherapy experience.

## Results

The ages of participants ranged between 22 and 55 years, with a median age of 32 years. Majority of them were females, Chinese, single, unemployed, completed the education of tertiary and above, stayed in purchased public housing, not hospitalized in the past year, and were without a comorbid physical problem. The number of psychotherapy sessions attended in the past 1 year ranged from 2 to 48 (median = 7.5). Table 2 provides a summary of the profile of clients.

**Table 2:** Characteristics of participants.

**Variable**		***N***
Age group	21–39	10
	40–65	5
Gender	Male	6
	Female	9
Ethnicity	Chinese	9
	Malay	4
	Indian	2
Marital status	Single	11
	Married	3
	Separated	1
Education	Secondary	2
	Vocational and diploma	6
	Tertiary and above	7
Housing type	Public (rented)	2
	Public (purchased)	10
	Private	3
Employment	Employed	4
	Not employed	11
Hospitalization in the past 1 year	Yes	4
	No	11
Physical health problems	Yes	7
	No	8
Type of mental disorder	Depression	7
	Mixed anxiety and depression	3
	Anxiety disorder	2
	Borderline personality disorder	1
	Not known	2

### Pre-psychotherapy

#### Predisposing Factors

As participants described their pathways or reasons to attend psychotherapy, several personal health-related beliefs and values, as identified by the authors, seemed to form their help-seeking behaviors. These included mental illness-related stigma in healthcare, lack of knowledge about psychotherapy as a treatment option, and preference for non-pharmacological treatment. The quotes representing each factor are presented in Supplementary Table 1.

##### Mental Illness-Related Stigma

Participants delayed help-seeking or were initially reluctant to attend psychotherapy session at a psychiatric institution for psychological problems for fear of being discriminated against or due to mental health stigma. The presence of social stigma created barriers to healthcare access and quality care [see Supplementary Table 1 (A1, A2)].

##### Lack of Knowledge About Psychotherapy

Participants expressed a lack of knowledge about the purpose and processes of psychotherapy. They were either unaware of psychotherapy as a viable option for their problems or unsure about the effectiveness of this treatment in solving their issues. Most participants only became aware and tried out psychotherapy without any expectations because they were being referred by another mental health professional or came to know about it when they read about it online [see Supplementary Table 1 (B1, B2)] .

##### Preference for Non-pharmacological Treatment

Patients either felt that medications were ineffective for them or were reluctant to embark on taking medications to manage their symptoms due to possible concerns of “addictiveness” or side effects. Therefore, they explored other non-pharmacological options such as psychotherapy as their preferred treatment of symptoms [see Supplementary Table 1 (C1, C2)].

#### Enabling Factors

The enabling factors explain the factors that facilitate or impede an individual to service use. Participants highlighted several hindrances to the utilization of psychotherapy services despite wanting to try or believe that psychotherapy is effective for them. These included inability to commit, affordability of service, and availability of resources (i.e., facilities and health personnel) in the community. The quotes representing each factor are presented in Supplementary Table 1.

##### Inability to Commit

While describing their experience with the utilization of psychotherapy, participants expressed the commitment issue as the main factor for not starting or continuing the therapy. The reasons cited include the lack of time, clash of schedules, inconvenience, or other personal concerns [see Supplementary Table 1 (D1, D2)] .

##### Affordability Issue

Despite being aware of the availability of psychotherapy services, some participants had concerns about continuing such services for a longer term as they felt it was too expensive. Some chose to engage psychotherapy services from public health providers instead of private sectors that were costlier [see Supplementary Table 1 (E1, E2)].

##### Service Unavailability

Few participants reported reasons related to the availability of resources, which hindered them from accessing or continuing psychotherapy service. These included the unavailability of psychotherapy tertiary care service offered in the preferred choice of a healthcare institution or the therapist of choice of a patient, as well as long waiting time [see Supplementary Table 1 (F1)].

#### Need Factors

This study identified both the perceived needs of patients (i.e., psychological symptoms and diagnosis) and the evaluated needs of mental health professionals (i.e., judgment about the health status of patients) as determinants that made participants seek and utilize psychotherapy service. The quotes representing each factor are presented in Supplementary Table 1.

##### Self-Perceived Mental Health Needs

The majority (i.e., 13/15) of participants were diagnosed with a mental disorder such as depression, anxiety, and borderline personality disorder. Participants reported the need to alleviate or cope with their underlying clinical symptoms and, hence, proceeded to seek psychotherapy service. Others felt that they just needed someone to talk to or to get support from due to the multiple psychological and social struggles that they were facing, and few insisted on seeing a psychotherapist despite being told it was not necessary by a health professional. Some participants also mentioned that they stopped going to the sessions when they felt better [see Supplementary Table 1 (G1)].

##### Professional Evaluation

Those who did not seek psychotherapy on their own were mainly referred to the service after presenting to a mental healthcare professional. They were prescribed psychotherapy by their consulting psychiatrist, during hospitalization or visit to the emergency services. Some participants went into psychotherapy due to their trust in the healthcare professionals or without even knowing what to expect from the service [see Supplementary Table 1 (H1)].

### During Psychotherapy

#### Therapy Process

Themes identified in this component pertain to common in-session experiences of the client and were contributed by the interplay of three broad elements, namely, the psychotherapist, the therapeutic modality, and the client her/himself. Participants also described the aspects of the sessions that were helpful or not helpful in improving their psychological well-being. The quotes representing each factor are presented in Supplementary Table 1.

##### The Attributes and Interactions of the Therapist Impact Alliance

Participants described mainly the positive qualities of their psychotherapists: “friendly,” “nice,” “gentle,” “non-judgmental,” “intelligent,” “good,” “concerned,” “well-informed,” “patient,” “attentive,” and “well-read,” with “understanding” being mentioned the most. These personal attributes of therapist appeared to strongly influence therapeutic alliance. The alliance was important to the therapeutic process and was also highly determined by the interaction of therapists with their clients. Understanding, caring, and accepting therapists were deeply valued by clients, while feeling unheard, misunderstood, and unappreciated challenged the alliance [see Supplementary Table 1 (I1, I2)].

##### The Application of Techniques by the Therapist Facilitates Alliance

Besides the personal attributes and communication skills of therapists, the significance of the expertise and modality of the therapist cannot be undermined and was also identified as important to therapeutic alliance and psychotherapy process. Most participants mentioned that they felt that their therapists were able to listen to them, understand them, and offer them good advice. A range of other specific techniques and strategies applied by the therapists during the in-session activities were also found to facilitate clients in identifying, viewing, and solving problems (Supplementary Table 2).

##### Match of Evidence-Based Treatment Modalities With the Preference of Clients

Evidence-based psychotherapy interventions were employed through either a single, integrative, or eclectic approach by therapists to match treatment to the individual and his/her psychiatric conditions. The commonly utilized forms of the evidence-based therapies based on the reports of participants were cognitive behavioral therapy (CBT) and mindfulness, while others included eye movement desensitization and reprocessing (EDMR), dialectical behavioral therapy (DBT), group therapy, exposure and response prevention (ERP), schema therapy, acceptance and commitment therapy (ACT), and psychodynamic therapy. While the majority found the assigned therapeutic approach helpful, others seemed to have their preferences and did not find certain intervention types to be helpful to them [see Supplementary Table 1 (J1, J2)].

##### Resistance of the Client in Psychotherapy

Despite the best efforts of psychotherapists, some clients failed to act in their best interests and engage fully in the therapeutic process. Such resistance impeded the motivation of the client and also interfered in treatment efficacy. Some participants were found to be reluctant to open up or discuss certain topics that were intrusive and distressing, particularly during the initial sessions or when therapists were new. Attending the sessions unprepared and unfocused was also a concern [see Supplementary Table 1 (K1)].

In addition, the success of the client in therapeutic outcome is usually dependent on doing homework or practicing strategies taught by the psychotherapist between sessions, in this study, the lack of motivation or effort led to non-compliance among few participants. They may have been either willing but were unable to complete the assigned task due to its length or difficulty, or simply unwilling to take it up at all [see Supplementary Table 1 (K2)].

##### Client Unaware of Treatment Plan

When asked about the kind of intervention they received or were receiving, some participants stated that they did not know the specific name of the therapy and that they were simply following through the therapy. While there was generally no complaint among these participants, few did express some unmet needs [see Supplementary Table 1 (L1, L2)].

### Post-psychotherapy

#### Therapeutic Outcomes

Several themes were identified in this section when participants described the perceived change in them from receiving psychotherapy. These were the reflections of the service efficacy and effectiveness or, in other words, therapeutic outcomes, which varied among participants. They included positive changes following therapy, sense of recovery not due to therapy, continued use of self-supporting strategies or online resources outside therapy, and problem coping or managing symptoms without therapist support. The quotes representing each factor are presented in Supplementary Table 1.

##### Positive Change Following Therapy

All the participants noted the beneficial effects of psychotherapy and experienced positive changes to varying extents. These improvements could be in the form of reduction in symptom severity or suicidal tendency, higher psychological well-being such as confidence and self-esteem, acquisition of better coping skills, or simply feeling better and supported after talk therapy [see Supplementary Table 1 (M1, M2)].

##### Recovery Beyond Effects of Therapy

Several participants cited that psychotherapy has its own limitations and could only help them to a certain degree. The previously experienced symptoms and struggles of clients improved as a result of the influence of events occurring outside of therapy or when the underlying issue got addressed but not due to the therapy. Others felt that their self-healing capacity or the intrinsic self is, if not more, important than the intervention itself for recovery [see Supplementary Table 1 (N1, N2)].

##### Engagement of Self-Supporting Strategies Outside Therapy

Therapists routinely imparted coping strategies and recommended online resources to their patients as part of their effort to integrate self-help into psychotherapy. Most clients cited the continued use of these self-supporting techniques and tools during waiting and maintenance stages. Participants described how these have helped them to cope with struggles or manage their symptoms effectively on their own when required at home or work [see Supplementary Table 1 (O1, O2)].

##### Problematic Coping in Absence of Therapist Support

Participants reported some form of reliance on their therapist during treatment phase. They expressed that they were unable to manage things on their own when they halted service after prolonged treatment or when they left the therapy room. Few participants had to resume psychotherapy despite having completed a previous course of treatment as they really needed someone to support them, with one even demanding for the same therapist [see Supplementary Table 1 (P1, P2)].

## Discussion

This study was a comprehensive study of the experience of clients with the service utilization of psychotherapy beginning from the pathway to care, followed by the therapy process, and lastly, response to therapy. Through in-depth interviews and qualitative analysis, the study derived themes associated with each phase of the service utilization of psychotherapy.

### Pathways and Reasons to Psychotherapy

Different reasons (i.e., indirect and direct) and obstacles to service access underlined themes identified in the pre-therapy stage and were found to complement the three factors, namely, predisposing, enabling, and need factors that were highlighted in Andersen's Healthcare Utilization Model (Andersen, 1995).

Studies have quantified and compared the strength of associations among the predisposing, enabling, and need factors with the use of psychotherapy. In a well-informed population with a high-quality insurance cover (i.e., low enabling factors), the use of psychotherapy was primarily associated with the clinical condition (i.e., need factors) rather than the sociodemographic status (i.e., predisposing factors) (Briffault et al., 2008). Hundt et al. found that predisposing and need factors were linked to the onset of the use of psychotherapy while enabling and need factors were linked to higher level use, and they also demonstrated that need factors were most strongly associated with the use of psychotherapy in veterans (Hundt et al., 2014). Findings from our study suggest that the predisposing factors such as mental health stigma and the lack of awareness of psychotherapy were significant barriers to the initial access of psychotherapy, but once overcome, these factors did not appear to influence the frequency of use. The enabling factors such as the lack of time, high treatment cost, and long wait time for the preferred therapist mainly impeded the increased or prolonged use of psychotherapy but did not affect the earlier decision of participants to embark on psychotherapy. In terms of need factors, mental health symptoms and struggles were cited when asked for the main concern for attending psychotherapy. Perceived recovery or the absence of health needs, as evidenced from the post-therapy experience, was also related to the discontinued engagement of psychotherapy.

### Psychotherapy Process and Therapeutic Outcomes

The experience of clients through the in-session activities and the therapy outcomes underlined themes in the during- and after-therapy stages, respectively. A large number of studies have been conducted into the process and outcome of psychotherapy from various lenses, with a substantial body of qualitative research focusing on the perspective of clients (Timulak, 2010; Timulak and McElvaney, 2013; Levitt et al., 2016). The study of the experience of therapy of the clients improves our understanding of the therapeutic process by shifting focus from the techniques, actions, and competencies of the therapists to include feelings, values, and attributes of the clients (Macran et al., 1999). A thematic review by Timulak revealed that, while clients valued factors relating to the client–therapist alliance during therapy, therapists were perceived to focus more on therapeutic gains (Timulak, 2010). Bachelor (2013) also found the views of the therapeutic alliance and therapeutic work between clients and therapists to differ such that, compared with therapists, clients tend to place greater emphasis on helpfulness, joint participation in therapy work, and negative signs of the alliance. The personal attributes (e.g., respectful, friendly, experienced, interested, open, warm, etc.) and the use of therapeutic techniques (e.g., supportive, understanding, exploration, reflection, accurate interpretation, affirming, etc.) of the therapist from a range of psychotherapy orientations were found to positively influence the development and maintenance of therapeutic alliance (Ackerman and Hilsenroth, 2003).

In fact, the findings from our study with respect to the “during service” period were consistent with the literature. First, some of the emergent themes (e.g., alliance between therapist and client, match between treatment modality and preference of client) involved various combinations of the three main aspects of psychotherapy, namely, client, therapist, and treatment modality, and did not involve only factors relating to the therapist or intervention alone. Second, the recollection of participants on the therapy process concentrated on how a range of characteristics and techniques of therapists similar to those described by Ackerman and Hilsenroth (Ackerman and Hilsenroth, 2003) was helpful to them and led to the positive therapeutic alliance, as well as on how the miscommunication of therapists ruined the alliance.

Therapeutic alliance essentially captures the interactive process between the client and the therapist and has been identified as the key variable in negotiating change or a reliable predictor of positive clinical outcomes in psychotherapy (Ackerman and Hilsenroth, 2003; Ardito and Rabellino, 2011). Besides therapeutic alliance, research into the effectiveness of therapy typically found other factors such as empathy, goal consensus and collaboration, the experience of therapists, therapy modality, and the level of motivation of the client to influence successful psychotherapy outcomes (Lynch, 2012; Wampold and Imel, 2015), all of which were consistent to the findings in our study. Previous studies on therapy outcomes were divided. The description of “good outcomes” among patients was found to cluster around four themes as follows: establishing new ways of relating to others, less symptomatic distress or change in behavioral patterns contributing to suffering, better self-understanding and insight, and accepting and valuing oneself (Binder et al., 2010). Other patients however, described themselves as not having improved through therapy and that therapy had not met their expectations (Radcliffe et al., 2018). All of our participants acknowledged some gains or positive changes, although few found therapy to have limited effectiveness and may not lead to full recovery.

Apart from the perspectives of clients, recent literature has reported that weekly therapy sessions appear to increase the rate of improvement compared with less frequent sessions although we have to keep in mind that this may vary according to setting, clinical population, and outcome measures (Robinson et al., 2020). On the contrary, studies have also reported that the number of psychotherapy sessions has less association with the therapeutic outcome (King, 2015; Flückiger et al., 2020). In our study, we did not examine this factor specifically, but it was observed that our participants who have had more sessions tended to report improvement. The effectiveness of the sessions was also reported by those who have had less than five sessions. We could have probably observed the expected trend with a larger sample.

### Expansion to Andersen's Behavioral Model

**Figure 2:**
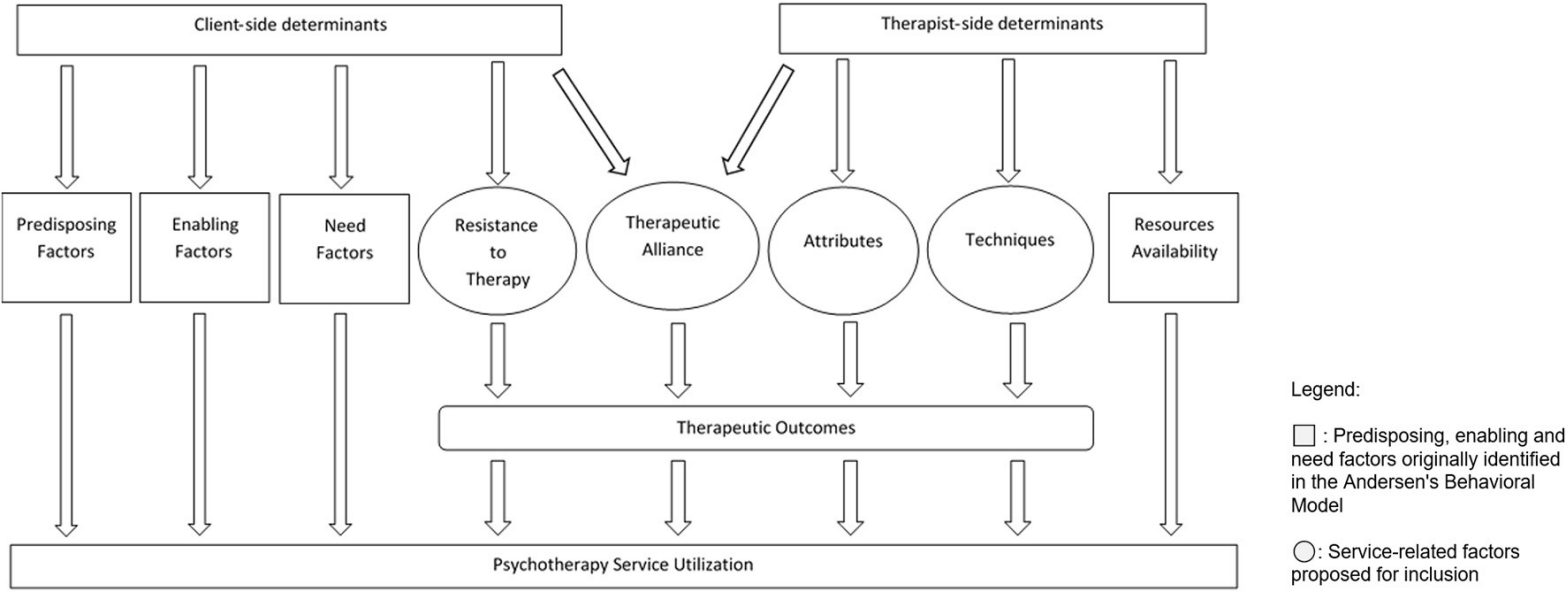
Proposed expanded framework for psychotherapy help-seeking behavior adapted from Andersen's Health Utilization Model.

Besides the numerous predisposing, enabling, and need factors as highlighted in Andersen's Behavioral Model, we also identified an additional component, i.e., service-related factors that we deemed to be important in understanding factors associated with the service utilization of psychotherapy (Figure 2). For example, clients' in-session and post-therapy experience may also impede or facilitate their decision to continue or complete treatment based on their account. Environmental obstacles, dissatisfaction with service, and lack of motivation for therapy were found to be the three most common reasons for premature termination of service (Anderson, 2016). Andersen proposed that the model offers flexibility in understanding health behaviors and researchers could add more factors to the original model, without disrupting its original structure to fit the purpose and nature of their research (Andersen, 1995). We have therefore proposed an expanded framework for the initial and continued use of psychotherapy service that incorporated the four abovementioned factors (i.e., predisposing, enabling, needs, and service-related factors) (Figure 2). The revised model also proposes the use of psychotherapy service to be a function of determinants due to both the client and the therapist. One limitation, however, will be the exclusion of components related to the health service policy and the healthcare system, which have been recognized as a criticism of the original Andersen's model (Andersen, 1995).

### Limitations of Study

There were several limitations in our study. First, as patients recruited for this study were mainly referred by their consulting therapists, they may not be open to discuss about the negative experiences they had with their therapists for fear that their therapists may learn about it despite being informed about the confidentiality and de-identification of the interview content. Second, the authors were unable to identify the distribution of the themes in the interviews as clients spent more time talking about what they found beneficial and not. Finally, the study was conducted among psychiatric patients attending psychotherapy in a discretionary health service (i.e., outpatient hospital service) setting, and hence, findings may not be generalized to all forms of psychotherapy services. Further studies are warranted to provide evidence for the proposed framework for the utilization of psychotherapy.

## Conclusion

This qualitative study may be the first to have obtained the in-depth experiences of psychotherapy of clients in Singapore, which enabled an evaluation of narratives from three phases, namely, pre-, during- and post-service encounters. The themes identified at the various stages concurred with those reported in other qualitative or quantitative studies. The study also expanded on Andersen's Health Service Utilization Model and proposed a promising framework to understand health behaviors and utilization relating to psychotherapy service. It also provides actionable information to address identified barriers to access and negative experiences or outcomes due to psychotherapy.

## Data Availability Statement

The raw data supporting the conclusions of this article will be made available by the authors, without undue reservation.

## Ethics Statement

This study involving human participants were reviewed and approved by National Healthcare Group Domain Specific Review Board. The patients/participants provided their written informed consent to participate in this study.

## Author Contributions

LSES and RS were involved in the conceptualization, data analysis, and drafted the manuscript. JV wrote up the protocol of the main study. SC and JV conducted the interviews. SC, RS, and LSES transcribed the audio files. LSES, RS, JV, and SC were involved in the coding process. MS was consulted for study design. HL, HA, and C-YT gave valuable inputs for the study and provided referrals for the interviews. All authors provided intellectual inputs and have approved the final manuscript.

## Conflict of Interest

The authors declare that the research was conducted in the absence of any commercial or financial relationships that could be construed as a potential conflict of interest.

## Publisher's Note

All claims expressed in this article are solely those of the authors and do not necessarily represent those of their affiliated organizations, or those of the publisher, the editors and the reviewers. Any product that may be evaluated in this article, or claim that may be made by its manufacturer, is not guaranteed or endorsed by the publisher.
