# Supplementary material for: A Qualitative Approach to Understanding the Holistic Experience of Psychotherapy Among Clients
Source: Front Psychol. 2021 Aug 6;12:667303. doi: 10.3389/fpsyg.2021.667303 (PMC8377233; doi:10.3389/fpsyg.2021.667303)
Supplement: Supplementary file 1 [file Table_1.docx]

Supplementary Table 1: Selected quotes representing participants opinions under the various themes

| Themes | Quotes |
| --- | --- |
| **1. Pre- Psychotherapy** | |
| **(a) Predisposing factors** | |
| (i) Mental illness-related stigma | ***A1****- “Actually, I wanted to switch totally to (another hospital) because I hate to be under Hospital A umbrella because I’m very traumatised” [Female, 49]*  ***A2****- “because I didn’t want to tell people I’m going Hospital A for therapy. It’s a bit hard…if I tell [people that] I’m going for therapy at Hospital A, people would view me as crazy or [that there is] something wrong with me. People will not be more accepting of me” [Male, 24]* |
| (ii) Lack of knowledge about psychotherapy | ***B1****- “but I didn’t know that I can just go to a doctor and explain that I need to speak to somebody because usually [you see a doctor only when] you are sick like [with] a flu or a cough right, I didn’t connect it”, [Female, 46]*  ***B2****- “……I don’t know, sometimes I also do wonder like if I really come for more frequent sessions, would my recovery be like accelerated kind or…”, [Female, 40]* |
| (iii) Preference for non-pharmacological treatment | ***C1****- “For now, there is three ways to solve for mental health. [First], medications which I am not so keen in even till now, [second], psychotherapy and [third], ECT (electroconvulsive therapy) which is the last choice…. so I do psychotherapy…”, [Male, 28]*  ***C2****- … actually the medicines were not working because the medicines are not targeted to stop your shoplifting, it is to calm you down and all that. I don’t need that. What I need is something else. So it is more of a therapy thing that I feel that the therapy really works because it works with your mood” [Female, 35]* |
| **(b) Enabling factors** | |
| (i) Inability to commit | ***D1****-“because minimally it (mindfulness therapy) takes like an 8-week program and I really can’t find the time because schedules are always [in the] evenings, 7-9 pm and those are like the most impossible times at home” [Female, 46]*  ***D2****- “there was some work [that] did not allow me to pursue my treatment, like you can imagine why. Whenever my boss gave me her “blessings” (referring to leave of absence to attend therapy sessions), my colleagues will not be very happy so I just put a stop until I hit another threshold and couldn’t handle any more so I continue. ”, [Female, 27]* |
| (ii) Affordability issue | ***E1****- “I did not stop because I thought I was good already. I stopped because it was expensive”, [Female, 46]*  ***E2****- “I was trying to find a therapist. But like because private therapist, they’re very pricey for me,” [Female, 22]* |
| (iii) Service unavailability | ***F1****- “Because Hospital A has this rule [that] I cannot have two therapists. ….. And then after all the therapy [sessions]… but he is not trained in schema therapy. So I need to change. And he tried to help find his colleagues, someone who he thinks is suitable for me, and it takes time. And this particular therapist is very busy, so I think it took about one to two months plus [before] I get to see this person”, [Male, 42]* |
| **(c) Need factors** | |
| (i) Self-perceived mental health needs | ***G1****- “I did express that I need a psychotherapist. After the initial talk, I think he (attending psychotherapist) feels very strongly that I don’t need…[but] I need help to regulate my thoughts and emotions especially over my parents’ death [which] affected me a lot... And I feel that my situation, financial situation, housing situation, everything was also a stress...then I did my reading on psychotherapy... Sometimes you can’t help yourself, it’s a bit weird. You need someone to like press the door…sometimes you can’t think very clearly. You need that kind of help”, [Male, 42]* |
| (ii) Professional evaluation | ***H1****- “I was also recommended to see a psychologist when I was seeing a private psychiatrist. And that was when I turned to this doctor (a psychotherapist with PhD) and I asked him about EMDR (eye movement desensitization and reprocessing). He felt that based on my background, CBT (cognitive behavioural therapy) is something that would be more appropriate for me. Yeah so that’s when he encouraged me to try out psychotherapy and I think if I didn’t have this level of trust in my psychiatrist, I would have been a lot less open to try out psychotherapy”,*  *[Male, 32]* |
| **2. During Psychotherapy** | |
| **Therapy process** | |
| (i) Therapist attributes and interactions impact alliance | ***I1****- “The therapist understands me. They are like supportive; they don’t judge me like my family does. And when I’m stuck and I can’t answer a question they don’t like press me for the answer. I remember this one time very clearly, one of my previous therapists I told her I felt really terrible that I had to pay somebody to listen to me and basically I meant her. She said that even though it’s true that this session was paid, she said that that didn’t diminish the, how to say, it didn’t diminish the value of the relationship. And that was something that really touched me - that she cared” [Female, 35]*  ***I2****- “I used to have one long time ago I call her stupid because she’s a psychologist from don’t know which hospital can’t remember now, uhm I didn’t like her. I call her stupid because that psychologist I asked her a question I know it’s personal but I thought I just wanted to get to know her, so I asked her whether she was married or how many children she has. And she said why do you want to know. Ok that really put me off so from then on I never wanted to see her again ok. Because…to me she’s like putting on airs it’s like what you know, why you want to know… I think people like that they are not fit to be psychologists, they should not be doing things like that because they don’t understand this thing called building rapport” [Female, 55]* |
| (ii) Match of evidence-based treatment modalities with clients’ preference | ***J1****- “I think CBT which I’m going through right now requires me as a patient to be very cognizant of I guess emotional triggers that happen to me. I think for a person whose default mechanism is an instinctive reaction to emotions, it gets particularly difficult to take a step back and to tap on something…which is why I mentioned also just now that if the stressor is a 5 out of 10, yes I can adopt most CBT techniques. If it is a 8 out of 10 then things that don’t require a lot of thinking but something that comes naturally like breathing exercises or certain visualisations is something that I would do” [Male, 32]*  ***J2****- “I found mindfulness more useful to me than CBT because…if I try to do CBT on myself, I will always out-argue myself. There’s always that angel devil thing going on in your head. So mindfulness therapy has been more useful to me because mindfulness therapy just works on accepting what you are feeling at that moment… you don’t worry about the future or the past if you’re doing it properly… (CBT) wasn’t helpful not because it was the therapist’s fault. I think it was me that I had a very critical inner voice. So because of that, CBT didn’t quite work”*  *[Female, 35]* |
| (iii) Client’s resistance in psychotherapy | ***K1****- “So for the first few therapy sessions, I wasn’t comfortable yet, so I didn’t really let go of everything [about myself] and I was just mainly talking about the things that are bothering me…it was when I finally made a list of the things, the steps or what I want to do in life. And then when I finally give it to her, and I guess she was quite happy about it. I think that is the thing she wanted from me all along – only focusing on myself”*  *[Female, 28]*  ***K2****- “homework does helps but the person must do. Uhm, but I suspect most people don’t like to do homework, because the homework takes a lot of effort. Like me, I am not working but I’m so lazy to do the homework, because it takes effort you see…” [Female, 55]* |
| (iv) Client unaware of treatment plan | ***L1****- “Only the previous therapist last time mentioned that ‘this is CBT that I’m teaching you but the recent one did not. So I suppose if they made it clear like what therapy they are using then it’s easier to read up on it and to practise it” [Female, 35]*  ***L2****- “they weren’t very clear about the kind of treatment plan that I’m going through. And I feel like a patient should have a right to know, not to interfere but just to have a knowledge of, a strong clarity of about they are doing? and at what point and what progress [I have made].” [Female, 27]* |
| **3. Post- Psychotherapy** | |
| **Therapeutic Outcomes** | |
| (i) Positive change following therapy | ***M1****- “my boyfriend tells me I have improved a lot already…I think I learnt to be more accepting. Yeah, I used to beat myself up a lot more when I have all those anxiety attacks. But now, I learnt to be gentler with myself….” [Female, 49]*  ***M2****- “any blind spot that I may overlook, [the therapist] gives me another perspective or view, in order that I can also manage my own condition…it helps me to understand my process of thinking and method of coping. And gives me viable options to try to see what can be. Because the more options you have, you have more ways you can contain the relapse”[Male, 28]* |
| (ii) Recovery beyond effects of therapy | ***N1****- “[I stopped psychotherapy] because I was ok, I didn’t feel depressed or I didn’t have any issues that I needed to clear so then I stopped the sessions. I mean [the concerns] were not really addressed by the therapist but sometimes it was resolved by myself or whatever, it just resolved by itself then I didn’t feel the need to come back” [Female, 35]*  ***N2****-"I feel like psychotherapy just helps with understanding the problems better… because psychotherapy is a lot of trial and error so it’s obviously going to take time but I think it’s the most they can do actually. And then I also feel that most of the things that has to do with maybe getting better mental health, it’s supposed to come from within…not just therapy itself. So I think both things have to work together” [Male, 22]* |
| (iii) Engagement of self-supporting strategies outside therapy | ***O1****- “They did take me through the process, because my therapist knows of some websites or they showed me websites about how to use mindfulness strategies. Then so like not only during the therapy sessions did I benefit from the mindfulness strategies, like they also give me websites to go through at home then or to go through at work.” [Male, 22]*  ***O2****- “she will recommend me some apps to use and of course I can select like the one that suits me best. So I would use that app to do breathing exercises to just help me fall asleep.”*  *[Female, 40]* |
| (iv) Problematic coping in absence of therapist support | ***P1****- “I did get better with the help of the exposure and response prevention (ERP) therapy, just that I’m still facing difficulties on how to cope with the symptoms better in camp and at home…” [Male, 22]*  ***P2****- “because I no longer lean outwards for social support (after attending psychotherapy), I think I’ve been a bit more isolated. I keep to myself a lot more…I’ve never thought about it in those terms but I just felt that there was an effect and my sessions currently being tapered off, it starts to worry me.” [Female, 27]* |
