# Supplementary material for: A Qualitative Approach to Understanding the Holistic Experience of Psychotherapy Among Clients
Source: Front Psychol. 2021 Aug 6;12:667303. doi: 10.3389/fpsyg.2021.667303 (PMC8377233; doi:10.3389/fpsyg.2021.667303)
Supplement: Supplementary file 2 [file Table_2.docx]

Supplementary Table 2: Examples of therapists’ specific techniques leading to successful psychotherapy outcomes

| Reflective questioning | *“she asked me what were your aspects in your life…so there’s partner, family and work etc and they are like our pillars. So then which are the ones that’s working and not working very well which is totally like terrible. Then I realize that it is actually not too bad…in majority…I should still be able to stand and not totally [unable to] function…it feels like though certain things or many things were still not sorted but at least in an aerial view I could say that my world is not so terrible”- Male,22* |
| --- | --- |
| Affirming client’s strength | *“during some of the sessions, my therapist actually when [going] through some exercises with me, I think it’s to help me like validate the positive [mind set] in me rather than to let my thoughts just like spiral in a negative fashion… when I’m down I just think of everything that brings me down. So it’s these kind of exercises do help me to a certain extent” – Female, 40* |
| Providing resources | *“almost after every session, she would also like email me some resources. Yeah, and because she knows my mom has dementia, so she would even send me resources on caregiving workshops and I remember like she even lent me a book on dementia. I mean like I don’t come for therapy every day, every other day, so those resources are really very helpful for like when I have pockets of time, I’ll just read them on my own and yeah, it’s like reminders to myself.” – Female, 40* |
| Values clarification | *“there’s a worksheet where she (the therapist) offers me like a set of values and then she’ll ask me like if to recognise whatever values that resonate with me most, the person I want to be” – Female, 22* |
| Empathic validation | *“sometimes it’s sort of like reaffirming what I feel. Like for example, let’s say if I say that somebody said this and that thing was a negative thing. Then she will say something like, “That must have been very hard to hear it. That must have been a very cruel thing to say.” So it’s sort of like she will mirror back what I would possibly have felt when that person said that thing. So it’s a kind of support in a way. Yeah it’s a kind of support in a way that somebody understands you”- Female, 35* |
| Cognitive restructuring | *“the current therapist, she tries to, she tries to put a balanced view into…yeah, she tries to give me a more balanced view of things. Like sometimes when I tell her I feel, like for example one thing I told her was I feel that I haven’t really progressed at all ever since my hospitalisation last year. And then she sort of like gave counter evidence that I have, I have actually progressed so I suppose it’s kind of helpful. It’s helpful because at least that’s, she helps to give a clearer analysis even though I may not see clearly…She didn’t give me any specific strategy for like changing my thought patterns or how I feel but she tries to sort of like tamper my views like when I have extreme views, she tries to bring up information that shows that it is not as extreme a situation as I think it is” – Female, 35* |
| Grounding | *“The therapist taught me how to ground myself when I am in the situation or [when] I got nightmares… it helps. But it’s a situational, for me my triggers are sometimes is, not all the times, babies crying so I have to ground myself with the things I like to do. So I have to find out what I like to do. When that time I hear babies crying I just go to that place and do the things I like to do” –Male, 24* |
| Self-disclosure | *“Example I got anxiety and then she shares her anxiety stories also…yeah that helps with, I don’t know, I feel more willing to open up more”- Male, 24* |
| Breaking down complex problems | *“my therapist, she actually does a really good job trying to break down the panic attacks and suicidal thoughts in like a more digestible format. So she actually knows how to separate different entities and make me understand it better“ – Male, 22* |
| Psychoeducation | *“she asked me to list a bunch of symptoms I had during panic attack…so she managed to find specific symptoms that I can counter first say- breathlessness…she actually tried to make me link that to exercise like if you’re running or if you’re swimming, you get breathless. So it’s not like you’re going to die, it’s just breathlessness so the way to counter it is to start breathing…she actually told me a few tips to do to avoid a panic attack escalating to something serious.” - Male, 22* |
| Attentional training | *“he drew a black circle with a red dot, so this is your focus, you have to think. He always tells me to remember this circle, so focus what you need to do. So before you do anything, calm yourself down first. He always [says], “calm yourself, calm yourself, don’t think about anything else around you, don’t think about it” – Female, 35* |
| Symptom monitoring | *“he used to give me this chart and tell me to circle the emotion. He had this emotion faces, so he will tell me to circle it. Each time you feel that, you cross it. How many times in that week you felt that way, you cross it. So every time I hand that over to him, he somehow knew how I was going through in that month” – Female, 35* |
